# Successful conservative management of diffuse cavernous hemangioma of the rectum

**DOI:** 10.1007/s13691-016-0262-x

**Published:** 2016-09-09

**Authors:** Katsuki Osaki, Yukiko Mori, Yoshinao Ozaki, Daisuke Yamaguchi, Akira Nozaki, Ikuo Aoyama, Masashi Kanai, Shigemi Matsumoto, Manabu Muto

**Affiliations:** 1 Kyoto University, Kyoto, Japan; 2 Department of Clinical Oncology, Kyoto University Hospital Cancer Center, 54 Shogoin Kawahara-cho, Sakyo-ku, Kyoto, 606-8507 Japan

**Keywords:** Cavernous hemangioma, Endoscopy, MRI, Rectum

## Abstract

Diffuse cavernous hemangioma of the rectum (DCHR) is a relatively rare disease. A 40-year-old man presented with long-standing lower abdominal discomfort and hematuria. At the time of hospitalization, his vital signs and hemoglobin level were normal. Colonoscopy showed markedly dilated blood vessels in the sigmoid mucosa, which was confirmed on magnetic resonance imaging and computed tomography as cavernous hemangioma. Without surgery, there have been no signs of progression of DCHR during a 3-year follow-up period.

## Introduction

Diffuse cavernous hemangioma of the rectum (DCHR) is a rare benign vascular tumor that is characterized by delayed diagnosis.

We report a case of a 40-year-old man who was admitted to our hospital due to long-standing lower abdominal discomfort and hematuria. He had no history of fever, abdominal pain, intermittent rectal bleeding, or weight loss. He also had no notable medical history or family history. His vital signs were normal and laboratory testing revealed a normal complete blood count without anemia.

**Fig. 1:**
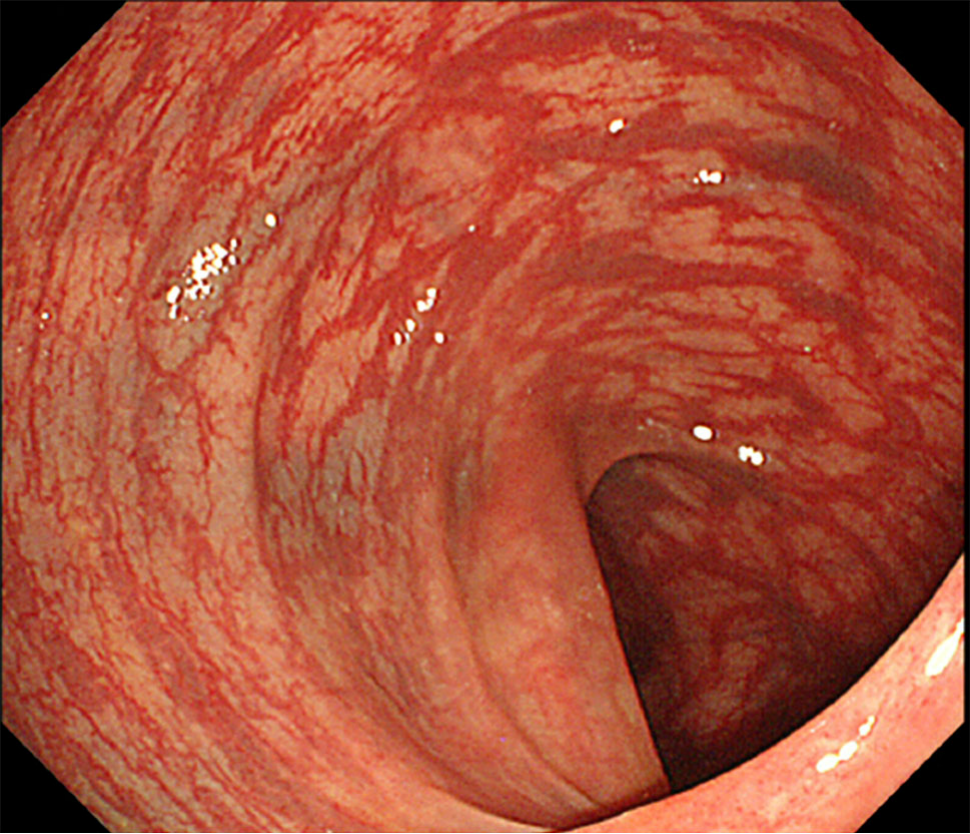
Colonoscopy findings. There are abnormally dilated blood vessels in the mucosa and submucosa of the sigmoid colon

**Fig. 2:**
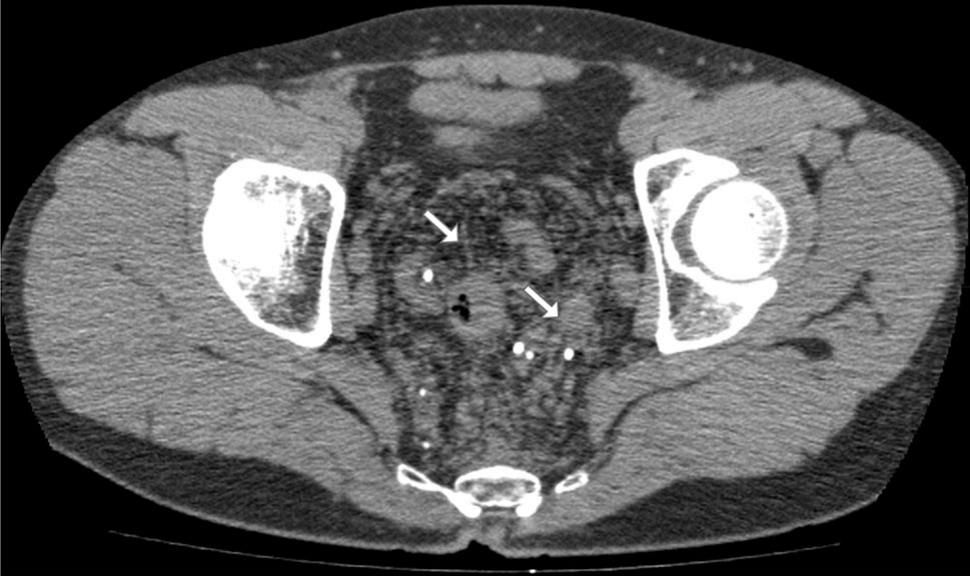
Computed tomography. The mucosal wall from the sigmoid colon to the rectum is thickened and surrounded by fatty tissue enhancement. Typical multiple calcified foci (*arrows*) are also seen

**Fig. 3:**
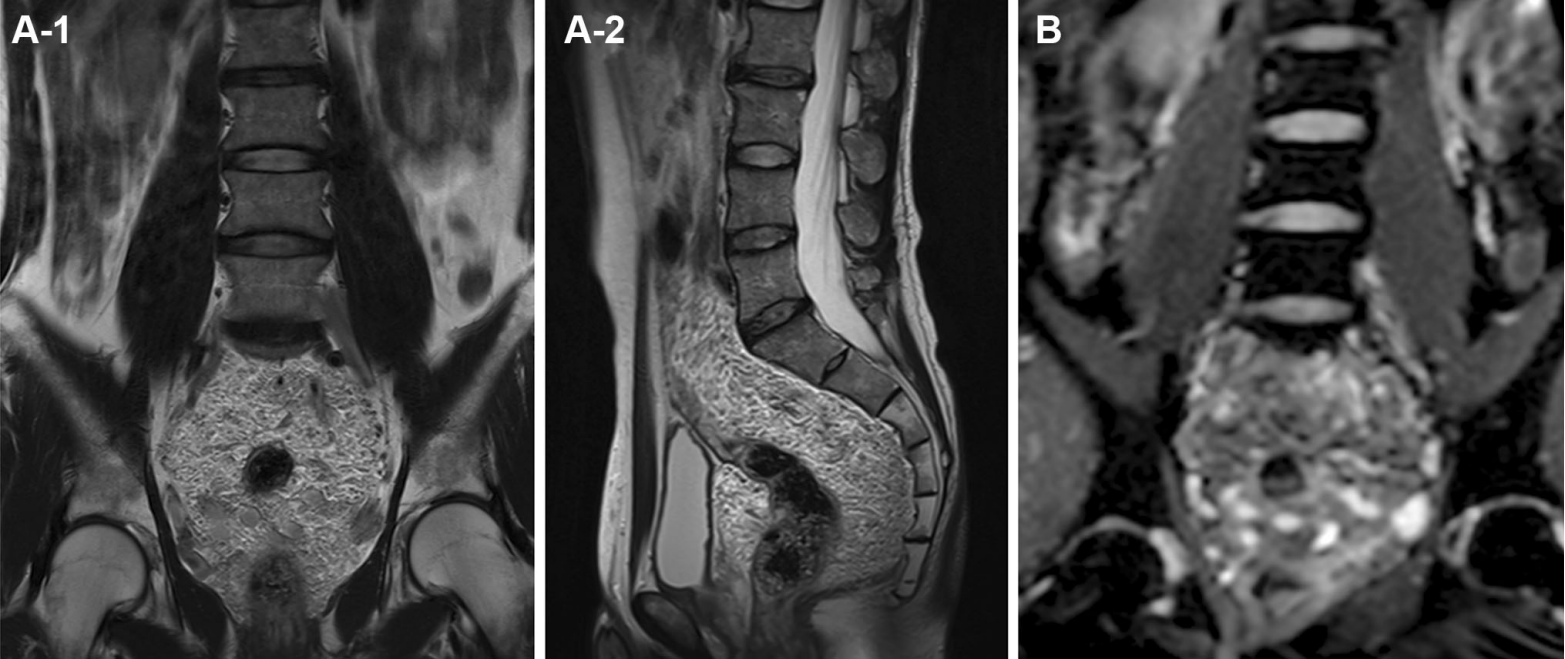
Magnetic resonance imaging. T2-weighted imaging (**a**1 coronal plane, **a**2 sagittal plane) shows thickened wall of the sigmoid colon and rectum and fatty tissue hyperplasia. Diffusion-weighted imaging (**b** coronal plane) show tortuous and dilated vessels in the fatty tissue

Colonoscopy showed markedly dilated blood vessels in the sigmoid mucosa (Fig. 1). Abdominal computed tomography (CT) revealed mucosal wall thickening from the sigmoid colon to the rectum, with hyperplasia of fatty tissue around the rectum. Venodilation with associated thrombosis in the dilated veins and multiple calcified foci associated with phleboliths were also detected (Fig. 2). Magnetic resonance imaging (MRI) also showed thickened wall from the sigmoid colon to the rectum, with tortuous and dilated vessels in the fatty tissue (Fig. 3). These findings were consistent with DCHR, and we decided to observe the patient. During a 3-year follow-up, there were no signs of progression of DCHR.

## Discussion

DCHR is a rare clinical condition, which mainly affects young adults. Since the first case of rectal hemangioma reported by Phillips in 1839, there have been only approximately 350 cases reported worldwide [1]. Recurrent painless rectal bleeding is the usual clinical symptom of DCHR and more than half of these patients have some degree of anemia [2]. However, DCHR may sometimes present as lower abdominal discomfort without the usual symptoms and may be misdiagnosed as hemorrhoids, colitis, polyposis, or rectal varicosities due to portal hypertension [1].

Although this disease presents with nonspecific symptoms, colonoscopy and abdominal CT and MRI can reveal specific findings that can serve as diagnostic clues; these typical and characteristic findings are irregularly thickened rectal wall and multiple calcified foci of pelvic phleboliths. In DCHR, biopsy is contraindicated because of the high risk for bleeding; the findings on CT or MRI are usually sufficient to make the diagnosis [2]. Multiple bluish submucosal varicosities and slight oozing are typical colonoscopic findings in DCHR [3, 4]. The presence of all these findings in this case confirmed the diagnosis.

Generally, complete surgical resection of DCHR is the only treatment and most cases are operated to control rectal bleeding [2]. Alternative therapies, such as sclerotherapy and selective embolization, cannot control rectal bleeding because DCHR originates from the dentate line and involves all layers of the rectal wall and the rectal mesentery. In previous reports, 33 of 43 DCHR cases underwent surgery (Table 1) [1, 5–9]. However, surgical resection of DCHR is complicated and can cause massive intraoperative bleeding; in a few cases, intermittent postoperative rectal bleeding was reported [1, 5]. In this case, there were no severe manifestations, such as bleeding or anemia, so we opted for observation without surgery. Fortunately, there have been no signs of progression of DCHR during a 3-year follow-up period and we were able to preserve the colon without invasive treatment.

**Table 1:** Previous reports on diffuse cavernous hemangioma of the rectum

	Number of patients	Surgery	Observation	Other
Nawa et al. [5]	18	14	4	0
Wang et al. [1]	17	14	0	3
Leal et al. [6]	2	2	0	0
Kandpal et al. [7]	2	0	0	2
Tan et al. [8]	2	1	1	0
Hasegawa et al. [9]	2	2	0	0
	43	33	5	5

In conclusion, colonoscopy, abdominal CT and MRI are useful to make a diagnosis of DCHR, and some case, especially those without a history of bleeding, could be followed-up efficiently without excessive invasive examinations.

## References

[1] Wang HT, Gao XH, Fu CG (2010). Diagnosis and treatment of diffuse cavernous hemangioma of the rectum: report of 17 cases. World J Surg.

[2] Yorozuya K, Watanabe M, Hasegawa H (2003). Diffuse cavernous hemangioma of the rectum: report of a case. Surg Today.

[3] Aylward CA, Orangio GR, Lucas GW (1998). Diffuse cavernous hemangioma of the rectosigmoid—CT scan, a new diagnostic modality, and surgical management using sphincter-saving procedures: report of three cases. Dis Colon Rectum.

[4] Lupetin AR (1990). Diffuse cavernous hemangioma of the rectum: evaluation and MRI. Gastrointest Radiol.

[5] Nawa T, Yoshihara H, Yamada Y (2008). A case of diffuse cavernous hemangioma of rectum in which fat-suppression MRI was useful for differential diagnosis. Gastroenterol Endosc.

[6] Leal RF, Ayrizono Mde L, Silva PV (2011). Laparoscopic-assisted bowel resection with construction of a colonic reservoir for cavernous hemangioma of the rectum: report of two cases. Tech Coloproctol.

[7] Kandpal H, Sharma R, Srivastava DN (2007). Diffuse cavernous haemangioma of colon: magnetic resonance imaging features. Report of two cases. Australas Radiol.

[8] Tan TC, Wang JY, Cheung YC (1998). Diffuse cavernous hemangioma of the rectum complicated by invasion of pelvic structures. Report of two cases. Dis Colon Rectum.

[9] Hasegawa H, Teramoto T, Watanabe M (1996). Diffuse cavernous hemangioma of the rectum: MR imaging with endorectal surface coil and sphincter-saving surgery. J Gastroenterol.

